# Equity effects of children’s physical activity interventions: a systematic scoping review

**DOI:** 10.1186/s12966-017-0586-8

**Published:** 2017-10-02

**Authors:** Rebecca E. Love, Jean Adams, Esther M. F. van Sluijs

**Affiliations:** Centre for Diet and Activity Research (CEDAR), MRC Epidemiology Unit, University of Cambridge School of Clinical Medicine, Box 285 Institute of Metabolic Science, Cambridge Biomedical Campus, Cambridge, CB2 0QQ UK

**Keywords:** Physical activity, Children, Interventions, Inequalities, Intervention-generated inequalities

## Abstract

**Background:**

Differential effects of physical activity (PA) interventions across population sub-groups may contribute to inequalities in health. This systematic scoping review explored the state of the evidence on equity effects in response to interventions targeting children’s PA promotion. The aims were to assess and summarise the availability of evidence on differential intervention effects of children’s PA interventions across gender, body mass index, socioeconomic status, ethnicity, place of residence and religion.

**Methods:**

Using a pre-piloted search strategy, six electronic databases were searched for controlled intervention trials, aiming to increase PA in children (6–18 years of age), that used objective forms of measurement. Screening and data extraction were conducted in duplicate. Reporting of analyses of differential effects were summarized for each equity characteristic and logistic regression analyses run to investigate intervention characteristics associated with the reporting of equity analyses.

**Results:**

The literature search identified 13,052 publications and 7963 unique records. Following a duplicate screening process 125 publications representing 113 unique intervention trials were included. Although the majority of trials collected equity characteristics at baseline, few reported differential effects analyses across the equity factors of interest. All 113 included interventions reported gender at baseline with 46% of non-gender targeted interventions reporting differential effect analyses by gender. Respective figures were considerably smaller for body mass index, socioeconomic status, ethnicity, place of residence and religion. There was an increased likelihood of studying differential effects in school based interventions (OR: 2.9 [1.2–7.2]) in comparison to interventions in other settings, larger studies (per increase in 100 participants; 1.2 [1.0 – 1.4]); and where a main intervention effect on objectively measured PA was reported (3.0 [1.3–6.8]).

**Conclusions:**

Despite regularly collecting relevant information at baseline, most controlled trials of PA interventions in children do not report analyses of differences in intervention effect across outlined equity characteristics. Consequently, there is a scarcity of evidence concerning the equity effects of these interventions, particularly beyond gender, and a lack of understanding of subgroups that may benefit from, or be disadvantaged by, current intervention efforts. Further evidence synthesis and primary research is needed to effectively understand the impact of PA interventions on existing behavioural inequalities within population subgroups of children.

**Trial registration:**

PROSPERO (PROSPERO 2016: CRD42016034020).

**Electronic supplementary material:**

The online version of this article (10.1186/s12966-017-0586-8) contains supplementary material, which is available to authorized users.

## Background

Health is unequally distributed across society. Evidence reveals social class gradients in health outcomes at every stage of the life course, with disadvantaged populations faring worse with regards to non-communicable disease risk prevalence and correspondingly life expectancy [1]. Many health behaviours are socially and economically patterned, playing a central role in shaping inequalities in population health outcomes through affecting the development of disease and overall quality of life [2, 3]. In developed and many developing countries, differences in physical activity behaviour across subgroups contribute to existing health inequalities, including stark, socially graded, differences in obesity prevalent across populations [4].

The benefits of engaging in regular physical activity during childhood and adolescence are well established, playing a critical role in promoting health and reducing future disease risk and mortality [5]. However, despite the breadth of well-documented health benefits [6–9], most children and adolescents do not meet global recommendations for physical activity and are not active enough to benefit their health [10, 11]. Following significant declines during the transition from childhood into adolescence, physical activity further declines into adulthood [12], with levels tracking across the lifespan [13–15]. Thus, differences in physical activity behaviour between subgroups of the adult population may develop during childhood. Accordingly, childhood is a critical time to intervene and change behaviour before patterns become entrenched for life [16].

The development of effective and sustainable interventions to increase physical activity in children has been identified by many governments and public health agencies as a key research priority for improving health outcomes [17]. However, the equity impacts of these interventions are unclear, with concern being raised regarding the possibility that even where interventions successfully improve overall behaviour across a population they also may inadvertently increase inequalities by not equally benefiting subgroups of individuals within the population [18, 19]. Differential effectiveness, frequently termed ‘intervention generated inequalities’, ensue when interventions provide greater benefit to one population group over another [20]. Such an effect is concerning when an intervention provides greater benefit to advantaged than disadvantaged groups. Evidence from evaluations of children’s physical activity interventions have revealed that inequalities are generated at multiple points throughout the intervention process including by differential provision of, and access to, interventions and resources [21], variation in uptake [22], differential intervention efficacy [23, 24], differential long term compliance [25] and differential response in evaluation [26]. While these evaluations of individual trials provide an indication of the potential for equity generating effects within children’s physical activity interventions, across the wider literature there is not a coherent overall understanding of the direction and size of effect across equity factors.

Despite the frequent use of systematic reviews for decision making, very few analyse or report equity effects [27]. Multiple, recent reviews have investigated the effectiveness of children’s physical activity interventions across varying settings [28–35], yet there is limited consideration for the differential effects of the included interventions. This has resulted in a lack of understanding of the characteristics and features of interventions that generate or reduce inequalities in children’s physical activity behaviour across population subgroups. In addition, it is possible our understanding of equity effects is biased due to underreporting of differential effects when statistical significance is not achieved. It is currently unknown whether there is sufficient consideration of differential effects across individual interventions to enable a full systematic review, and furthermore whether trials report appropriate data to allow for retrospective analysis of the question. Given this lack of clarity we conducted this review in a scoping manner to map out the existing state of the literature.

The purpose of this scoping review was to assess the availability of evidence for differential effects of children’s physical activity interventions and investigate the characteristics of interventions that study differential effectiveness. The collation of evidence through this systematic scoping review will be valuable in providing an overview of the literature, with an aim of identifying where evidence gaps exist to direct future research.

## Methods

With an aim of identifying research gaps and mapping out the existing literature by examining the extent and nature of research, this review was conducted as a scoping review. A literature search was conducted to identify relevant published controlled trials designed to promote physical activity in children 6–18 years of age in school, community, home or health-care based settings. Searches were conducted in six electronic databases (ERIC, EMBASE, SCOPUS, PsycINFO, Medline, SPORTDiscus) in May 2016. All sources were searched with a pre-piloted search strategy with no restrictions by publication year, geographic location, ethnicity or other socio-demographic indicators. Searches were limited to manuscripts available in English. The search strategy as used in Medline is included in Additional file 1: File S1*.* The review protocol was registered with PROSPERO (CRD42016034020).

### Inclusion criteria

The search strategy was designed to retrieve controlled trials (Study design) of single or multicomponent interventions in the school, home, health-care or community environment (Intervention), aimed at increasing school-aged children and adolescent’s levels of physical activity (Population), with a minimum intervention or normal control group (Control), and objectively assessed physical activity at baseline and follow-up (Outcome). The full inclusion and exclusion criteria are outlined in Table 1. These inclusion criteria were based on existing knowledge of the literature base demonstrating the presence of numerous controlled trials [33], using objective forms of physical activity measurement [32], within the population of interest. As self-reported activity is also likely to be differentially biased [36], we established restrictive inclusion criteria, while conducting the review in a scoping manner to map out the availability of evidence contained within the trials.

**Table 1:** Intervention inclusion & exclusion criteria

	Included	Excluded
Population	• Children and adolescents, 6–18 years of age at baseline	• Pre-school populations of children (5 years of age and younger) • Children selected on the basis of having a specific disease or special needs
Intervention	• Single or multicomponent interventions aimed at increasing physical activity in the school, home or community environment	• Interventions with a duration less than 4 weeks
Study design	• Controlled or randomised controlled trials (cluster or individual) with a minimal intervention or control group	• Trials comparing two active intervention arms
Outcomes	• Objectively measured physical activity across the whole day at baseline and follow-up (E.g. accelerometer, pedometer heart rate)	• Subjectively measured physical activity outcomes (E.g. self-report questionnaires) • Assessments where follow-up measurements were not collected in the same children as at baseline • Interventions examining physical activity for only part of the day (E.g. recess or breaktime)
Publication type	• Peer reviewed journal article	• Conference abstract, study protocol, report, dissertation, book
Publication year	• Any year	• N/A
Language	• English	• All other languages

### Intervention screening and selection

Primary article titles identified following de-duplication of the initial search were manually screened and those clearly outside the review criteria discarded. The abstracts of the remaining citations that passed the initial title screening were independently reviewed and compared to the inclusion criteria to determine if retrieval of the full primary study was needed for further examination. The initial literature searches and scanning stages (title, abstract) were conducted by one reviewer (RL). A 15% random sample was double checked at each stage (EvS). The full text screening was performed in duplicate by two authors (RL, EvS). At the full text phase, related and pre-identified reviews on the same topic were scanned for missing trials [29, 31–33]. All discrepancies were resolved through discussion amongst the research team.

### Supplementary searches for associated publications

For each trial that met the inclusion criteria, steps were taken to retrieve all associated publications to ensure that equity analyses reported separately to the main intervention effect paper were captured. To find associated publications for each included trial, subsequent searches were performed using trial names and registration numbers. Additionally, forward citation tracking on Google Scholar was used to screen and identify additional trial publications that referenced the main effect paper included in this review.

### Data extraction

For each trial that met the inclusion criteria, intervention characteristics and covariates were extracted using a pre-established data extraction form and Microsoft Excel. At each stage of the review process, all data was managed using Mendeley Reference Manager. Data extraction was performed in duplicate (RL, JA). The extracted data included trial name, journal of main intervention effect paper and year of publication, study population and size, setting, baseline descriptive data, equity data collected at baseline, intervention type (physical activity only or multi-behaviour intervention), intervention targeting (by gender, BMI (body mass index), ethnicity, socioeconomic status (at the individual, school or community level), place of residence and religion), intervention effects across all outcomes, differential effect analyses and the methods utilized to investigate differential effects (by subgroup or interaction analysis). ‘Subgroup analyses’ were classified as the evaluation of treatment effects by subgroups of participants defined at baseline by an equity characteristic, while ‘interaction analyses’ were identified as the use of an overall statistical test to directly compare differences in intervention effects across subgroups [37].

Equity data and analyses were considered across PROGRESS-Plus, a framework created to ensure explicit consideration for the multiple intersecting factors that affect health equity within research and intervention design [38]. Differential effects were considered across all factors outlined by the PROGRESS-Plus framework applicable to a child population: gender, socioeconomic status (SES), ethnicity, place of residence, and religion [39]. SES data and analyses were further classified by whether SES had been measured at the family, school or community level to give an indication of how SES was conceptualised in this context. In addition, BMI was included as an additional equity factor of particular relevance in the context of physical activity interventions in consideration of substantial evidence indicating it is patterned by SES, geographic area and ethnicity [40–42]. Other factors included in the PROGRESS-plus framework (occupation, social capital) were not considered relevant within a child population and excluded. All discrepancies in data extraction were resolved through discussion amongst the research team. As per standard practice for scoping reviews, methodological quality assessment of included interventions was not performed [43].

### Analysis

Graphical and narrative methods were used to summarize the results. Subsequently, logistic regressions analyses were performed to determine if certain intervention or study characteristics influenced the likelihood of reporting of differential effects. Intervention and study characteristics of interest included as exposure variables in logistic regression models were journal impact factor, country of origin, intervention setting, participants’ ages, sample size and whether or not positive main intervention effects were reported. Outcomes comprised of whether or not any equity effects were studied, and whether or not gender equity effects were studied. No other equity characteristics were considered frequently enough to allow for further analysis. Univariable models were run for each exposure-outcome pair.

## Results

**Fig. 1:**
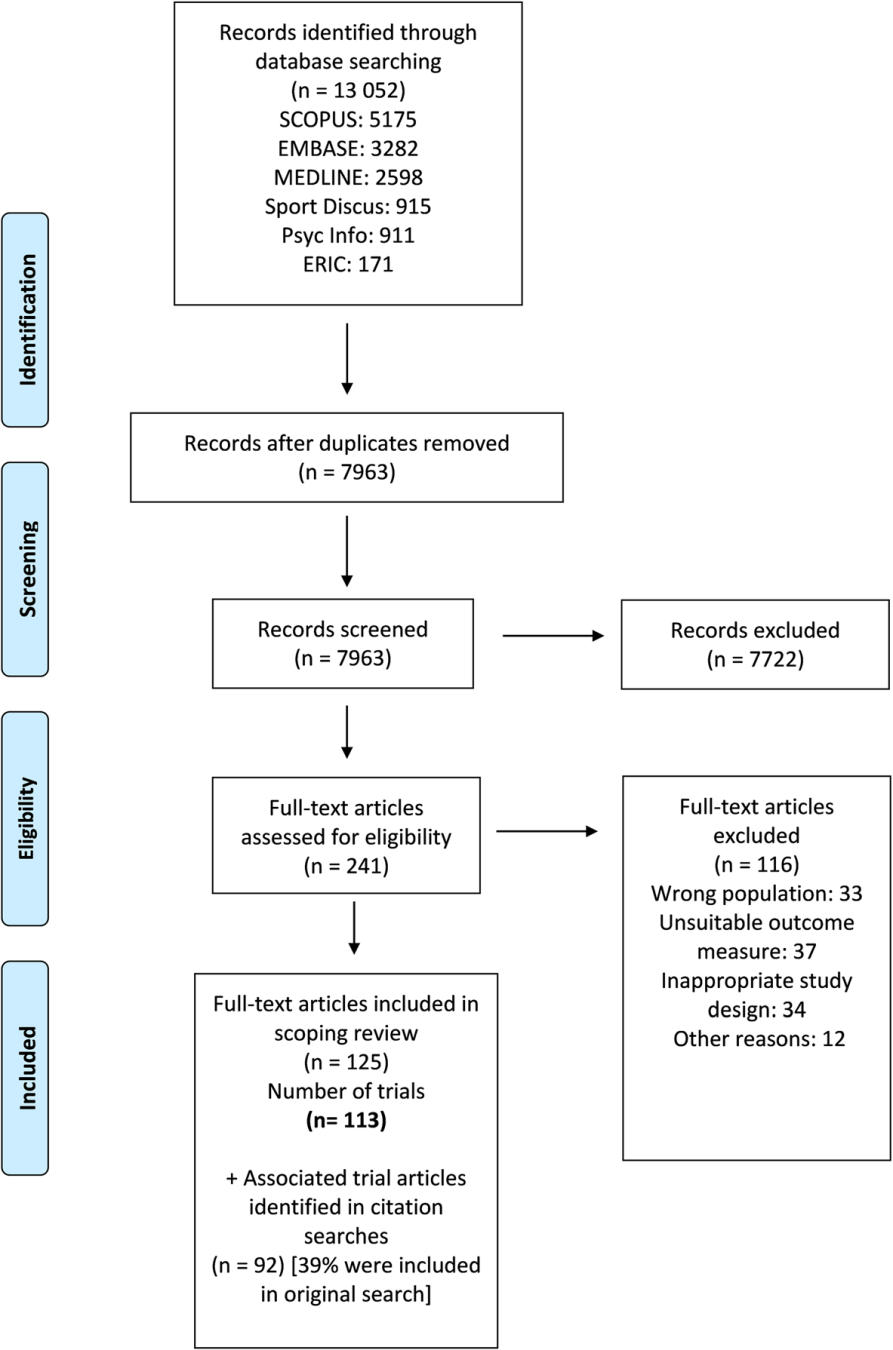
PRISMA flow diagram

Figure 1 outlines the search and screening process. The database search resulted in the identification and retrieval of 13,052 records, including 7963 unique records after removal of duplicates. Following title and abstract scanning, 241 potentially relevant articles were screened in full text. Ensuing assessment against the inclusion criteria led to inclusion of 125 publications representing 113 intervention trials (See Additional file 1: File S2). Citation and trial registration number searches identified an additional 92 associated publications, of which 39% had appeared in the original database search. The reference lists of included trials and associated publications are included as additional files (See Additional file 1: Files S3 and S4, respectively).

### Characteristics of included interventions

The characteristics of included trials are outlined in Table 2. The majority of the 113 included trials were conducted in Europe (40%), followed by North America (35%) and Australasia (20%). Of the remaining 5%, 4 were conducted in Asia and 2 in South America. Only 3 were conducted in Low and Middle Income Countries (Mexico [44]; Ecuador [45]; Turkey [46]). Forty-two percent of trials were targeted solely at physical activity behaviour change, while 58% were targeted at multiple health behaviours: primarily a combination of diet and physical activity. Of the included trials, 74% had intervention components that took place in school-based settings, 56% in home-based settings, 30% in community-based settings and 3% in healthcare-based settings.

**Table 2:** Characteristics of included trials (*N* total = 113)

Age at baseline (mean, SD)	10.3 (2.8)
Sample size (mean, SD)	267.3 (385.1)
Study location	n (%)
Australasia	23 (20%)
Europe	44 (40%)
North America	40 (35%)
Other	6 (5%)
Country income level	
High-income	110 (97%)
Low and middle income	3 (3%)
Study setting^a^	
School-based	84 (74%)
Community-based	34 (30%)
Home-based	63 (56%)
Healthcare-based	3 (3%)
Study type/behaviour	
PA-only	66 (58%)
Multi-behaviour	47 (42%)
Reported equity characteristic at baseline^a^	
Gender	113 (100%)
BMI	86 (76%)
Ethnicity	60 (53%)
SES	60 (53%)
Place of residence	3 (3%)
Religion	0 (0%)
Targeted by^a^	
Gender	24 (21%)
BMI	22 (19%)
Ethnicity	19 (17%)
Individual SES	0 (0%)
School SES	17 (15%)
Community SES	19 (17%)
Place of residence	3 (3%)
Religion	0 (0%)
Reported a main effect:	
By any outcome	102 (90%)
By objectively measured physical activity	75 (66%)

Categories marked with a ^a^ are not mutually exclusive

The mean sample size of included trials was 267 (SD: 385.1), ranging from 18 to 3010 participants. The average age of participants at baseline ranged from 6 to 16.5 years of age, with a mean of 10.3 years (SD: 2.3). Of the 113 included interventions, 21% were targeted specifically by gender, while 19% were targeted by BMI and 17% by ethnic groups. In addition, a number of interventions were targeted by school (15%) and community level SES (17%). Of all included trials, 90% reported a main intervention effect on any outcome while 66% reported a main intervention effect on objectively measured physical activity.

### Differential effect analyses

**Fig. 2:**
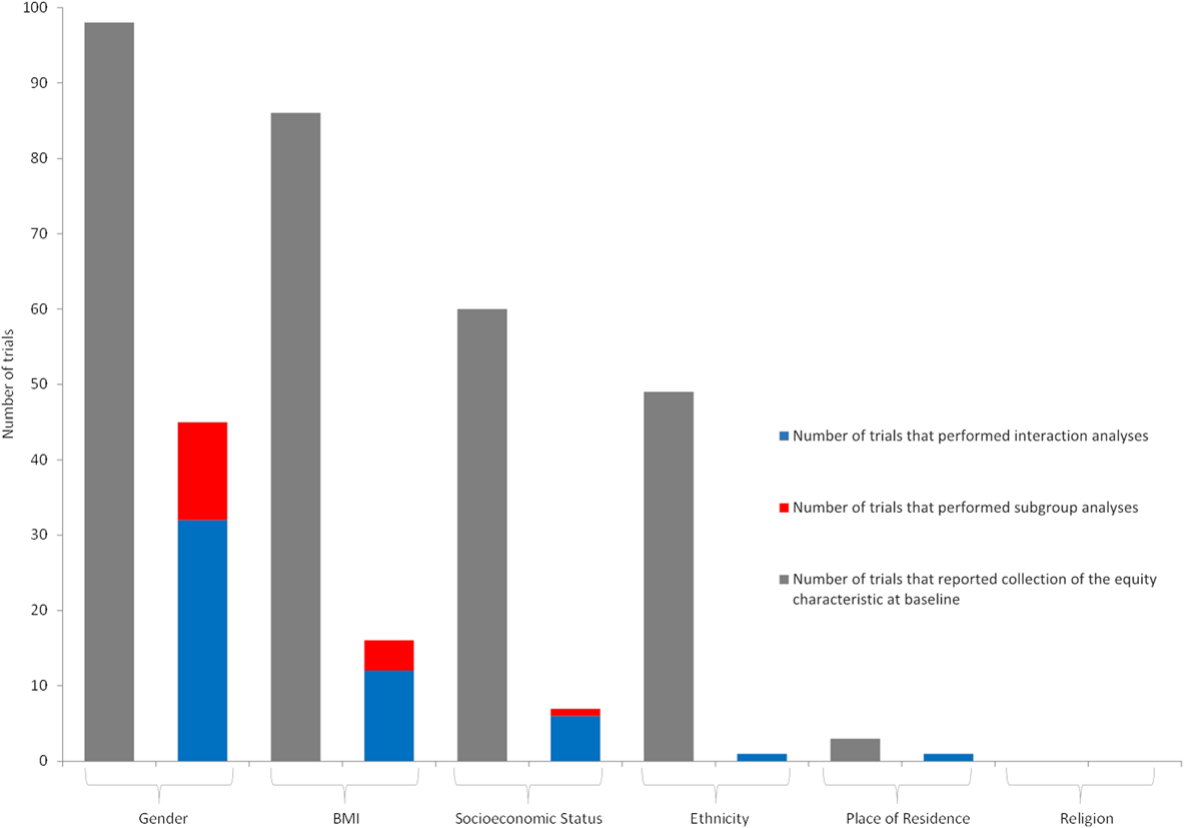
Total number of trials that reported each equity characteristic of interest at baseline and number of which reported differential analyses by subgroup and interaction analysis. Trials targeted by each equity characteristic are not included in the figure

Figure 2 presents the number of included trials that captured equity data at baseline, and the number that subsequently conducted equity analyses. Of the 98 interventions not targeted by gender, all reported gender data, with 45 of the 98 (46%) exploring differential effects by gender through subgroup (71%) or interaction analysis (29%). Across the remaining equity characteristics, differential effects were explored substantially less frequently. Of the 86 included interventions with reported BMI data, 16 (19%) reported differential effects. Only 7 of the 60 (12%) trials with reported SES data, 1 of the 49 (2%) with reported ethnicity data and 1 of 3 (33%) with reported place of residence data documented exploration of differential effects. Of the 70 equity analyses reported, most were performed by subgroup analysis (74%) with considerably fewer by interaction analysis (26%).

### Factors predicting differential analyses

Table 3 highlights the characteristics of differential effect analyses by each equity characteristic. Logistic regression models indicated that significantly more is known about equity in the context of school-based interventions in comparison to other contexts (home, community and health-care based) (See Table 4). Studies investigating school-based interventions were 2.9 times (95% CI: 1.2–7.2) more likely to report differential effects by any factor and 4.5 times (95% CI: 1.5–13.2) more likely to report differential effects by gender.

**Table 3:** Differential analyses across all equity characteristics

Differential analysis	By gender /Total	BMI	Ethnicity	SES	Place of residence	Religion
Total number of non-targeted studies	98	113	102	113	112	113
Location:						
Australasia	6/18 (33%)	3/23 (13%)	0/23 (0%)	1/23 (4%)	0/23 (0%)	0/23 (0%)
European	25/40 (63%)	4/44 (9%)	1/43 (2%)	5/44 (11%)	1/43 (2%)	0/44 (0%)
North American	10/34 (29%)	7/40 (18%)	0/30 (0%)	1/40 (3%)	0/40 (0%)	0/40 (0%)
Other	4/6 (67%)	1/6 (17%)	0/6 (0%)	0/6 (0%)	0/6 (0%)	0/6 (0%)
Publication year:						
2004 & earlier	2/6 (33%)	0/8 (0%)	0/4 (0%)	0/8 (0%)	0/8 (0%)	0/8 (0%)
2005–2009	10/18 (56%)	2/21 (10%)	0/21 (0%)	0/21 (0%)	0/21 (0%)	0/21 (0%)
2010–2014	25/54 (46%)	12/63 (19%)	1/57 (2%)	6/63 (10%)	0/62 (0%)	0/63 (0%)
2015 & above	9/20 (45%)	1/21 (5%)	0/20 (0%)	1/21 (5%)	1/21 (5%)	0/21 (0%)
PA only or multi-behaviour intervention:						
PA only	27/58 (47%)	7/66 (11%)	0/64 (0%)	2/66 (3%)	1/46 (2%)	0/66 (0%)
Multi-behaviour	19/40 (48%)	8/47 (17%)	1/37 (3%)	5/47 (11%)	0/66 (0%)	0/47 (0%)
Intervention setting:						
Home based	23/55 (42%)	8/63 (13%)	0/54 (0%)	5/63 (8%)	1/62 (2%)	0/63 (0%)
School based	41/74 (55%)	11/84 (13%)	1/78 (1%)	6/84 (7%)	1/83 (1%)	0/84 (0%)
Community based	12/28 (43%)	6/34 (18%)	0/29 (0%)	2/34 (6%)	1/33 (3%)	0/34 (0%)
Health-care based	3/3 (100%)	0/3 (0%)	0/3 (0%)	0/3 (0%)	0/3 (0%)	0/3 (0%)
Age, children vs adolescents:						
Children	34/78 (44%)	11/86 (13%)	0/78 (0%)	5/86 (6%)	1/86 (1%)	0/86 (0%)
Adolescents	12/20 (60%)	4/27 (15%)	1/24 (4%)	2/27 (7%)	0/26 (0%)	0/27 (0%)
Main intervention effect, any outcome:						
Main effect	46/90 (51%)	12/102 (12%)	1/92 (1%)	6/102 (6%)	1/101 (1%)	0/102 (0%)
No main effect	0/8 (0%)	3/11 (27%)	0/10 (0%)	1/11 (9%)	0/11 (0%)	0/11 (0%)
Main intervention effect, objectively measured PA:						
Main effect	39/70 (56%)	10/75 (13%)	1/71 (1%)	5/75 (7%)	0/74 (0%)	0/75 (0%)
No main effect	7/21 (33%)	5/38 (13%)	0/31 (0%)	2/38 (5%)	1/38 (3%)	0/38 (0%)

Note: Denominators are not consistent as each row is restricted to the number of non-targeted trials, separated by each equity characteristic

**Table 4:** Logistic regression models exploring factors predicting analysis of differential effects

	OR (95% confidence interval) of reporting differential effects by any equity characteristic (*n* = 113)	OR (95% confidence interval) of reporting differential effects by gender (*n* = 98)
Australasia vs all others	0.4 (0.1–2.1)	0.2 (0.0–1.8)
European vs all others	1.1 (0.2–5.8)	0.8 (0.1–5.1)
North American vs all others	0.4 (0.1–2.1)	0.2 (0.0–1.3)
PA only (1) or multi-behaviour intervention (0)	0.7 (0.3–1.5)	1.1 (0.5–2.4)
Home-based vs all others	0.6 (0.3–1.4)	0.6 (0.3–1.3)
School-based vs all others	**2.9 (1.2–7.2)**	**4.5 (1.5–13.2)**
Community based vs all others	0.9 (0.4–2.0)	0.8 (0.3–2.0)
Health-care based vs all others	Not enough variation to run	Not enough variation to run
Age (Child under 12 (0), Adolescent 13–18 (1))	1.4 (0.6–3.2)	1.6 (0.6–4.2)
Sample Size (Per increase in 100 participants)	**1.2 (1.0–1.4)**	**1.2 (1.0–1.4)**
Journal impact factor	1.1 (0.9–1.3)	1.1 (0.9–1.2)
Reported a main intervention effect on any outcome	2.5 (0.6–9.8)	Not enough variation to run
Reported a main intervention effect on objectively measured physical activity	**3.0 (1.3–6.8)**	**3.6 (1.3–9.5)**

Significant findings are highlighted in bold

As expected, due to differences in statistical power, an increase in sample size was associated with an increased odds ratio of conducting differential effect analysis (OR: 1.2, 95% CI: 1.0–1.4, per additional 100 participants). Country of origin, intervention type, age and journal impact factor were not significantly associated with reporting of differential effects.

Regression models indicated that a main intervention effect on objectively measured physical activity was associated with subsequent exploration of differential effects by equity subgroups (3.0 (95% CI: 1.3–6.8)). When restricted to exploration of differential effects by gender this likelihood increased to a odds ratio of 3.6 (95% CI: 1.3–9.5).

## Discussion

To the best of our knowledge, this is the first review to provide a comprehensive overview of available evidence on consideration of equity effects in the children’s physical activity literature. We have revealed a scarcity of consideration for equity. Despite all included trials collecting at least one equity characteristic of interest at baseline, a limited number reported investigating analyses of differential effectiveness. When reported, differential effect analyses were primarily concentrated on gender, with substantially fewer focusing on BMI, SES, ethnicity, place of residence or religion. The failure of authors to report equity analyses (despite having data available with which to do this) reinforces a lack of understanding of, and importance given to, intervention generated inequalities.

The wider health literature supports these findings, with reviews of both smoking interventions and universal school-based behavioural interventions indicating similar rates of equity analyses, with accompanying calls for more routine testing of differential effects [47, 48]. Similar to these results, analyses within the adult physical activity intervention literature have found that despite researchers commonly measuring equity characteristics at baseline, differential effect analyses are infrequently reported in trial evaluations [49, 50]. Likewise, when reported, analyses are mostly confined to gender, with considerably less attention given to other equity characteristics.

The lack of equity focus identified in this review is surprising considering the widespread public health policy focus on inequality [27, 51, 52]. Despite overarching policy goals, in practice we have a very limited understanding of the potential for inequality generating effects from current intervention efforts. As a research community we are not accumulating the evidence policy makers need to deliver on objectives and targets for the development and implementation of interventions that effectively reduce health inequalities. Considering the state of the evidence and paucity of data, we recommend and echo prior calls for the conduct and reporting of differential effect analyses [50]. However, we acknowledge the financial and resource requirements of running sufficiently large trials powered to detect a main intervention effect, let alone differential effects between subgroups. To tackle these critical questions, we encourage a coordinated effort towards fewer, high-quality, large trials, adequately powered to address questions of differential effectiveness. Continuing to amass evidence solely to address the question of overall effectiveness will only propagate our current level of understanding and limit the evidence base from progressing.

We acknowledge the potential generation of false negative results as a consequence of subgroup and interaction analyses with inadequate statistical power [53–55]. While it is encouraging that included interventions with a larger sample size were more likely to perform differential effect analyses, we do not specifically know what proportion of the 70 differential effect analyses (74% by interaction and 26% by subgroup analysis) were adequately powered. Considering that many trials focus on recruiting sufficient participants to detect differences in effect between intervention arms [56], it is crucial that each analysis is interpreted sensibly, and the credibility of the analyses carefully scrutinized independently against established criteria [37, 57–59]. Guidelines generally advise conducting a small number of differential effect analyses, that are pre-specified and based on strong theory, adjustment for multiple testing is considered, and that reporting indicates if analyses were pre-planned or performed post-hoc. Unfortunately, previous evidence has indicated that differential effect investigations by subgroup analyses are often not pre-specified in protocols, and even when they are 90% deviate from the described plan [60]. When possible, interaction analyses should be preferentially performed as these provide a more direct test of differences in effect [61]. Considering the possibility that reporting of differential effect analyses is dependent on the achievement of statistical significance at a *p* ≤ 0.05 level, we need to continue moving towards required pre-specification in protocols and analyses plans, and the enforcement of reporting of any deviations and accompanying rationales in trial publications by reviewers and journals. Alongside this evidence is the proposition that authors may be particularly likely to explore subgroup analyses if they did not find a main intervention effect. Encouragingly, this hypothesis was not supported within this review, with trials that found a main intervention effect being significantly more likely to conduct differential effect analyses in comparison to those that did not.

Girls are well known to be on average less active then boys [62, 63]. This observation is likely influencing the focus on assessment of differential intervention effects by gender. Moreover, compared to gender, SES and ethnicity are challenging to accurately measure within populations of children and adolescents. Evidence has shown difficulties in the conceptualization of SES, and inconsistencies in the relevance of tangible measures of education, occupation and income in relation to children’s perceived SES [64]. Additionally, when parental questionnaires are utilized to help overcome these differences new challenges arise. Evaluations indicate that the completion of parental questionnaires and consent forms is socially patterned with factors including poor literacy levels among low income parents affecting the return of signed consent forms [65]. Furthermore, gender is generally equally distributed across participant samples and study groups. In comparison, ethnicity and SES often end up considerably skewed towards the majority within that specific context, since intervention trials are frequently implemented within a restricted region of schools and neighbourhoods. These differences in distributions may result in an increased likelihood of gender being adequately powered for differential effect analyses in comparison to the remaining equity characteristics. It is likely that these issues contribute to the differences and patterns identified in these analyses.

There is growing evidence that certain subgroups such as girls, children with disabilities, and those from minority ethnic groups and low SES families or neighbourhoods have lower levels of physical activity than their counterparts [63, 66–72], which contribute to associated and apparent health inequalities [73]. In response, a multitude of interventions tailored to the characteristics of high-risk subgroups have been developed [31], as evidenced in this review with more than a third of included trials targeted by at least one equity factor and a subset of these targeted by multiple equity characteristics. The comparative effectiveness of targeted vs. non-targeted interventions is largely unknown as the interventions evaluated differ substantially. Although subgroups of high-risk children may benefit from an intervention targeted directly at them, public health benefits in terms of physical activity and health outcomes may be limited in the absence of a population approach. Rose’s theory of disease prevention suggests that it is more efficient to utilize a universal program approach that works to shift the entire population distribution of a risk factor then focus exclusively on a high-risk subgroup through a targeted intervention [74]. Analyses of differential effects in response to one universal intervention revealed greater benefits to girls and inactive children, but also significant benefits to boys and those already active [75]. This suggests that a gender-targeted approach in this case may have disregarded a subgroup also able to benefit. While it is likely that the optimum population preventative strategy incorporates a tiered combination of both targeted and universal approaches, the optimal balance for the greatest impact on behaviours and disease risk at maximal cost-effectiveness is unclear. Given this state of the evidence, we highlight the concurrent need for research of the comparative effectiveness of interventions targeted specifically at population subgroups and those that are universally targeted. It is critical these efforts work to understand the comparative effectiveness (i.e. behaviour change in girls within a female targeted vs a universal intervention) while considering the lack of effect within the non-targeted subgroup (i.e. loss of any effect in boys from the dissemination of a female targeted intervention).

This scoping review has multiple strengths, including the systematic searches, duplicate review methods, and the consideration of a wide range of evidence. As is inherent within a review, this work is limited by reporting and quality within the included primary studies. Due to the nature of the review as a scoping exercise to map out available evidence, we did not look at the reporting and analysis of interaction and subgroup effects in a detailed manner. We also recognize the limitations inherent in combining a heterogeneous set of intervention studies with varying aims and implemented across a variety of settings. We further acknowledge the intrinsic challenges in the use of SES, due to the fact it is measured at multiple levels (individual, home, community SES), with each captured by numerous indicators (parental education/occupation, asset based indicators, free-school meals). As appropriate for a scoping review, we are unable to draw conclusions regarding the extent of differential effectiveness in children’s physical activity promotion efforts. However, the results indicate that there may be sufficient data available (published and unpublished) for a more in-depth exploration of differential effectiveness, either through meta-analyses or pooling of primary data. This may need to be performed within a more homogeneous subset of studies, and take the operationalization of varying indicators into consideration.

## Conclusion

There is a widespread lack of knowledge of the equity effects of children’s physical activity interventions. Despite often collecting relevant information at baseline, most controlled trials do not report analyses of differences in intervention effect. More evidence is needed to effectively understand how current intervention efforts are affecting existing behavioural inequalities across population subgroups of children, while being mindful of the tension with statistical constraints. Understanding the characteristics of interventions that generate differential effects has important implications for directing future research and intervention development. As governments and international health organizations increasingly advocate the need for equity focused evidence to inform population interventions addressing health inequalities, there needs to be action to ensure that intervention evaluations and systematic reviews consider and address these equity effects.

## Additional file

Additional file 1: File S1. Medline Search Strategy. **File S2.** Characteristics of included intervention trials. **File S3.** References of Included Trials. **File S4.** Associated publications. (DOCX 48 kb)

## Abbreviations

BMI: Body mass index
PA: Physical activity
SES: Socioeconomic status
