# Supplementary material for: Equity effects of children’s physical activity interventions: a systematic scoping review
Source: Int J Behav Nutr Phys Act. 2017 Oct 2;14:134. doi: 10.1186/s12966-017-0586-8 (PMC5625682; doi:10.1186/s12966-017-0586-8)
Supplement: Additional file 1: File S1. — Medline Search Strategy. File S2. Characteristics of included intervention trials. File S3. References of Included Trials. File S4. Associated publications. (DOCX 48 kb) [file 12966_2017_586_MOESM1_ESM.docx]

**Additional file 1**

**File S1. Medline Search Strategy**

| 1. (child* or children or childhood or kids or adolescen* or "young person*" or "young people" or teen* or youth* or boy* or girl* or juvenile).ti,ab.  2. exp child/  3. exp adolescent/  4. 2 or 3  5. (child or adolescent).ti,ab.  6. 1 or 5  7. ("physical* activ*" or "physical activity" or sport* or cycling or bicycling or bicycle* or walk* or "physical education" or "physical training" or exercis* or "energy expenditure" or danc* or "physical inactivity" or "physical fitness" or lifestyle or "active travel" or commut* or "aerobic fitness").ti,ab.  8. exp motor activity/  9. exp sports/  10. exp exercise/  11. exp physical exertion/  12. exp "physical education and training"/  13. 8 or 9 or 10 or 11 or 12  14. (motor activity or sports or exercise or physical exertion or "physical education and training").ti,ab.  15. 7 or 14  16. ("clinical trial" or "control* trial" or controlled or randomi#ation or randomised or randomized or randomization or randomly or randomisation or rct or "randomi#ed controlled trial*" or "randomised controlled trial" or "randomized controlled trial" or "cluster randomized controlled trial" or "group-randomized controlled trial" or "randomized controlled study" or "randomised controlled study" or "random* sample" or trial* or evaluation or effect* or control* or cluster or intervention).ti,ab.  17. exp randomized controlled trial/  18. exp clinical trial/  19. exp randomized controlled trials as topic/  20. exp clinical trial as topic/  21. 17 or 18 or 19 or 20  22. (randomized controlled trial or clinical trial or randomized controlled trials as topic or clinical trial as topic).ti,ab.  23. 16 or 22  24. ("case study" or "case report" or "abstract report" or letter).ti,ab.  25. exp letter/  26. exp historical article/  27. exp case report/  28. 25 or 26 or 27  29. (letter or historical article or case report).ti,ab.  30. 24 or 29  31. 23 not 30  32. (accelerometer or accelerometry or accelerometers or accelerometer-assessed or "counts per minute" or CPM or triaxial or Actigraph or Yamax or Actiheart or Omron, sensewear or caltrac or walk4life or ideea or actireg or lifecorder or tritrac or genea or stepwatch or actical or actiwatch or rt3 or activpal or actimarker or dynaport or CSA or MTI or pedometer or "heart rate" or pedometry or pedometers or uniaxial or actigraphy or undimensional or "objectively measur*" or "SenseWear Pro2 Armband" or "motion sensor data" or "activity monitor" or MVPA).ti,ab.  33. exp monitoring, ambulatory/  34. exp actigraphy/  35. 33 or 34  36. (monitoring, ambulatory or actigraphy).ti,ab.  37. 32 or 36  38. 6 and 15 and 31 and 37  39. 6 and 15 and 31 and 37  40. limit 39 to english language |
| --- |

**File S2. Characteristics of included intervention trials**

| **Included Intervention Trials**  (Author (year) is included if trial has no name) | **Author & Publication Year** | **Country of implementation** |
| --- | --- | --- |
| Aburto (2011) | Aburto et al. 2011 | Mexico |
| Andrade (2014) | Andrade et al. 2014 | Ecaduor |
| Backlund (2011) | Backlund et al. 2011 | Sweden |
| Baranowski (2011) | Baranowski et al. 2011 | USA |
| Baranowski (2012) RCT | Baranowski et al. 2012 | USA |
| The Memphis Girls’ health Enrichment Multi-site Studies (GEMS) Pilot trial | Beech et al. 2003 | USA |
| Mebane on the Move Intervention | Benjamin Neelon et al. 2015 | USA |
| Challenge! Intervention | Black et al. 2010 | USA |
| Sport for LIFE intervention | Breslin et al. 2012 | UK |
| Copenhagen School Child Intervention Study | Bugge et al. 2012 | Denmark |
| Pathways | Caballero et al. 2003 & Going et al. 2002 | USA |
| Comprehensive school physical activity program (CSPAP) | Carson et al. 2014 | USA |
| Chen (2010) | Chen et al. 2010 | USA |
| Web ABC study | Chen et al. 2011 | USA |
| The Supporting Children's Outcomes using Rewards, Exercise, and Skills intervention (SCORES) Cluster RCT | Cohen et al. 2015 | Australia |
| Beat the Street physical activity intervention | Coombes et al. 2016 | UK |
| The Family Project | Coppins et al. 2011 | UK |
| Boston Active School Day policy | Cradock et al. 2014 | USA |
| Crouter (2015) | Crouter et al. 2015 | USA |
| D'Haese (2015) | D’Haese et al. 2015 | Belgium |
| The nutrition and enjoyable activity for teen (NEAT) girls study | Dewar et al. 2013 & Dewar et al. 2014 & Lubans et al. 2012 | Australia |
| Dimitriou (2011) | Dimitriou et al. 2011 | Greece |
| Apps for IMproving FITness and Increasing Physical Activity Among Young People: The AIMFIT Pragmatic Randomized Controlled Trial | Direito et al. 2015 | New Zealand |
| Physical Activity Across the Curriculum (PAAC) | Donnelly et al. 2008 | USA |
| Dudley (2010) | Dudley et al. 2010 | Australia |
| The Healthy Homework pilot study | Duncan et al. 2011 | New Zealand |
| Fit4fun Pilot Study | Eather et al. 2013 | Australia |
| Fit4fun group randomized controlled trial | Eather et al. 2013 | Australia |
| It's child's play: A cluster randomised controlled trial | Engelen et al. 2013 | Australia |
| Erwin (2011) | Erwin et al. 2011 | USA |
| Eyre (2016) | Eyre et al. 2016 | UK |
| FATaintPHAT | Ezendam et al. 2012 | Netherlands |
| Children's Health, Activity and Nutrition: Get Educated! (CHANGE!) Project | Fairclough et al. 2013 | UK |
| Finkelstein (2013) | Finkelstein et al. 2013 | Singapore |
| Ford et al. (2012) | Ford et al. 2012 | UK |
| SWITCH what you Do, View and Chew | Gentile et al. 2009 | USA |
| Lifestyle triple P | Gerards et al. 2015 | Netherlands |
| Interactive Multimedia for Promoting Physical Activity (IMPACT) in Children | Goran et al. 2005 | USA |
| GreatFun2Run | Gorley et al. 2009 & Gorley et al. 2011 | UK |
| Gortmaker (2012) | Gortmaker et al. 2012 | USA |
| Graves (2010) | Graves et al. 2010 | UK |
| HEalth in Adolescents (HEIA) study | Grydeland et al. 2013 | Norway |
| The Rope Skipping 'STAR' Programme | Ha et al. 2015 | Hong Kong |
| Haerens (2007) | Haerens et al. 2006 & Haerens et al. 2007 | Belgium |
| Hands (2011) | Hands et al. 2011 | Australia |
| Harder-lauridsen (2014) | Harder-lauridsen et al. 2014 | Denmark |
| Fit 'n' fun dudes program (2009) | Hardman et al. 2009 | UK |
| Fit 'n' fun dudes program (2011) | Hardman et al. 2011 | UK |
| Walking School Bus - Nebraska | Heelan et al. 2009 | USA |
| The Sports, Play, and Recreation for Youth (SPARK) program | Herrick et al. 2012 | USA |
| Fit for Life Boy Scouts Program | Jago et al. 2006 | USA |
| The Bristol Girls Dance Project Feasibility Trial | Jago et al. 2012 | UK |
| Action 3:30 (Jago et al. 2014) | Jago et al. 2014 | UK |
| The Bristol Girls Dance Project | Jago et al. 2015 | UK |
| Active for Life Year 5 (AFLY5) | Kipping et al. 2014 | UK |
| The Memphis Girls’ health Enrichment Multi-site Studies (GEMS) | Klesges et al. 2010 | USA |
| KISS school-based physical activity program | Kriemler et al. 2010 & Meyer et al. 2014 | Switzerland |
| Laukkanen (2015) | Laukkanen et al. 2015 | Finland |
| Lee (2012) | Lee et al. 2012 | Taiwan |
| Lubans & Morgan (2008) | Lubans & Morgan. 2008 | Australia |
| Program X intervention | Lubans et al. 2009 & Lubans et al. 2010 | Australia |
| Physical Activity Leaders (PALS) | Lubans et al. 2011 & Lubans et al. 2012 | Australia |
| MacConnie (1982) | MacConnie et al. 1982 | USA |
| School-Community Partnerships: a Cluster RCT | Madsen et al. 2013 | USA |
| Energy Balance 4 Kids with Play RCT | Madsen et al. 2015 | USA |
| Magnusson (2011) | Magnusson et al. 2011 | Iceland |
| Maloney (2008) | Maloney et al. 2008 | USA |
| STOPP Cluster RCT | Marcus et al. 2009 | Sweden |
| McManus (2008) | McManus et al. 2008 | Hong Kong |
| McMinn (2012) | McMinne al. 2012 | UK |
| Meinhardt (2013) | Meinhardt et al. 2013 | Switzerland |
| Walking School Bus - Texas (Mendoza et al. 2011) | Mendoza et al. 2011 | USA |
| Couch Potatoes to Jumping Beans | Mhurchu et al. 2008 | New Zealand |
| The Healthy Dads, Healthy Kids community randomzied controlled trial (2011) | Morgan et al. 2011 | Australia |
| The Healthy Dads, Healthy Kids community randomzied controlled trial (2014) | Morgan et al. 2014 | Australia |
| The Great Activity Programme | Morris et al. 2013 | UK |
| Children, parents and pets exercising together (CPET) | Morrison et al. 2013 | UK |
| Action Schools! BC | Naylor et al. 2008 | Canada |
| Healthy School Start Study | Nyberg et al. 2015 | Sweden |
| Healthy School Start Study II | Nyberg et al. 2016 | Sweden |
| Y-PATH Intervention (O'Brien et al. 2013) | O’Brien et al. 2013 | Ireland |
| BOUNCE (Behavior Opportunities Uniting Nutrition, Counseling, and Exercise) trial | Olvera et al. 2010 | USA |
| Promoting Lifestyle Activity for Youth (PLAY) Intervention | Pangrazi et al. 2003 | USA |
| Sigue la Huella intervention | Pardo et al. 2014 | Spain |
| PACE+ for adolescents | Patrick et al. 2006 | USA |
| Prochaska (2004) | Prochaska et al. 2004 | USA |
| Reza (2014) | Reza et al. 2014 | Turkey |
| Reznik (2015) | Reznik et al. 2015 | USA |
| Encouraging Activity to Stimulate Young (EASY) Minds' programme | Riley et al. 2015 | Australia |
| The EASY Minds pilot RCT | Riley et al. 2015 | Australia |
| Robbins (2012) | Robbins et al. 2012 | USA |
| Roemmich (2004) | Roemmich et al. 2004 | USA |
| Roemmich (2012) | Roemmich et al. 2012 | USA |
| Swwitch play intervention | Salmon et al. 2008 | Australia |
| Schodielf (2005) | Schodielf et al. 2005 | Australia |
| The Nereu Program | Serra-Paya et al. 2015 | Spain |
| HYPPE - Helping Youth Pursue Physical Activity and Exercise (Shore et al. 2014) | Shore et al. 2014 | USA |
| Sigmund (2012) | Sigmund et al. 2012 | Czech Republic |
| Active Teen Leaders Avoiding Screen-time (ATLAS) cluster randomized controlled trial | Smith et al. 2014 | Australia |
| Minnesota GEMS Pilot Study | Story et al. 2003 | USA |
| Straker (2013) | Straker et al. 2013 | Australia |
| Physical Activity 4 Everyone | Sutherland et al. 2013 & Sutherland et al. 2013 | Australia |
| The APPLE project | Taylor et al. 2006 | New Zealand |
| The Mulicomponent SPACE Study: A Cluster RCT | Toftager et al. 2014 | Denmark |
| The MOVE Project | Tymms et al. 2016 | UK |
| The prevention of dietary- and lifestyle-induced health effects in children and infants (IDEFICS) intervention (IDEFICS) | Verbestel et al. 2015 | 8 European Countries (Belgium, Cyprus, Estonia, Germany, Hungary, Italy, Spain and Sweden) |
| UP4FUN pilot intervention (2012) | Verloigne et al. 2012 | Belgium |
| Verstraete (2007) | Verstraete et al. 2007 | Belgium |
| lauft' trial | Vivien Suchert et al. 2015 | Germany |
| Wells (2014) | Wells et al. 2014 | USA |
| Wilson (2002) | Wilson et al. 2002 | USA |
| Wilson (2005) | Wilson et al. 2005 | USA |
| Active by Choice Today (ACT) | Wilson et al. 2011 | USA |

**File S3: References of Included Trials**

Aburto NJ, Fulton JE, Safdie M, Duque T, Bonvecchio A, Rivera JA. Effect of a school-based intervention on physical activity: Cluster-randomized trial. Med Sci Sports Exerc. 2011;43:1898–906.

Andrade S, Lachat C, Ochoa-Aviles A, Verstraeten R, Huybregts L, Roberfroid D, et al. A school-based intervention improves physical fitness in Ecuadorian adolescents: A cluster-randomized controlled trial. Int J Behav Nutr Phys Act. 2014;11:153

Bäcklund C, Sundelin G, Larsson C. Effects of a 2-year lifestyle intervention on physical activity in overweight and obese children. Adv Physiother. 2011;13:97–109..

Baranowski T, Abdelsamad D, Baranowski J, O’Connor TM, Thompson D, Barnett A, et al. Impact of an active video game on healthy children’s physical activity. Pediatrics. 2012;129:e636–42.

Baranowski T, Baranowski J, Thompson D, Buday R, Jago R, Griffith MJ, et al. Video game play, child diet, and physical activity behavior change: A randomized clinical trial. Am J Prev Med. 2011;40:33–8.

Beech BM, Klesges RC, Kumanyika SK, Murray DM, Klesges L, McClanahan B, et al. Child- and parent-targeted interventions: the Memphis GEMS pilot study. Ethn Dis. 2003;13 1 Suppl 1:S1-53.

Benjamin Neelon SE, Namenek Brouwer RJ, Østbye T, Evenson KR, Neelon B, Martinie A, et al. A Community-Based Intervention Increases Physical Activity and Reduces Obesity in School-Age Children in North Carolina. Child Obes. 2015;11:297–303.

Black MM, Hager ER, Le K, Anliker J, Arteaga SS, DiClemente C, et al. Challenge! Health promotion/obesity prevention mentorship model among urban, Black adolescents. Pediatrics. 2010;126:280–8.

Breslin G, Brennan D, Rafferty R, Gallagher AM, Hanna D, G. B, et al. The effect of a healthy lifestyle programme on 8-9 year olds from social disadvantage. Arch Dis Child. 2012;97:618–24.

Bugge A, El-Naaman B, Dencker M, Froberg K, Holme IMK, McMurray RG, et al. Effects of a three-year intervention: the Copenhagen School Child Intervention Study. Med Sci Sports Exerc. 2012;44:1310–7.

Caballero B, Clay T, Davis SM, Ethelbah B, Rock BH, Lohman T, et al. Pathways: a school-based, randomized controlled trial for the prevention of obesity in American Indian schoolchildren. Am J Clin Nutr. 2003;78:1030–8..

Chen JL, Weiss S, Heyman MB, Cooper B, Lustig RH. The efficacy of the web-based childhood obesity prevention program in Chinese American adolescents (Web ABC study). J Adolesc Heal. 2011;49:148–54.

Carson RL, Castelli DM, Pulling Kuhn AC, Moore JB, Beets MW, Beighle A, et al. Impact of trained champions of comprehensive school physical activity programs on school physical activity offerings, youth physical activity and sedentary behaviors. Prev Med (Baltim). 2014;69 S:S12–9.

Chen JL, Weiss S, Heyman MB, Lustig RH. Efficacy of a child-centred and family-based program in promoting healthy weight and healthy behaviors in Chinese American children: A randomized controlled study. J Public Health (Bangkok). 2010;32:219–29.

Cohen KE, Morgan PJ, Plotnikoff RC, Callister R, Lubans DR, K.E. C, et al. Physical activity and skills intervention: SCORES cluster randomized controlled trial. Med Sci Sports Exerc. 2015;47:765–74.

Coombes E, Jones A. Gamification of active travel to school: A pilot evaluation of the Beat the Street physical activity intervention. Heal Place. 2016;39:62–9.

Coppins DF, Margetts BM, Fa JL, Brown M, Garrett F, Huelin S. Effectiveness of a multi-disciplinary family-based programme for treating childhood obesity (the Family Project). Eur J Clin Nutr. 2011;65:903–9.

Cradock AL, Barrett JL, Carter J, McHugh A, Sproul J, Russo ET, et al. Impact of the Boston Active School Day policy to promote physical activity among children. Am J Health Promot. 2014;28 3 Supplement:S54–64.

Crouter SE, de Ferranti SD, Whiteley J, Steltz SK, Osganian SK, Feldman HA, et al. Effect on physical activity of a randomized afterschool intervention for Inner City Children in 3rd to 5th grade. PLoS One. 2015;10:e0141584–e0141584.

De Bock F, Genser B, Raat H, Fischer JE, Renz-Polster H. A participatory physical activity intervention in preschools: A cluster randomized controlled trial. Am J Prev Med. 2013;45:64–74.

Dewar DL, Morgan PJ, Plotnikoff RC, Okely AD, Batterham M, Lubans DR. Exploring changes in physical activity, sedentary behaviors and hypothesized mediators in the NEAT girls group randomized controlled trial. J Sci Med Sport. 2014;17:39–46.

Dewar DL, Morgan PJ, Plotnikoff RC, Okely AD, Collins CE, Batterham M, et al. The nutrition and enjoyable activity for teen girls study: A cluster randomized controlled trial. Am J Prev Med. 2013;45:313–7.

D’Haese S, Van Dyck D, De Bourdeaudhuij I, Cardon G. Effectiveness and feasibility of lowering playground density during recess to promote physical activity and decrease sedentary time at primary school. BMC Public Health. 2013;13:1154.

Dimitriou M, Michalopoulou M, Gourgoulis V, Aggelousis N. Participation in community-based sport skills learning programmes, physical activity recommendations and health-related fitness for children in Greece. Sport Sci Health. 2011;6:93–102.

Direito A, Jiang Y, Whittaker R, Maddison R. Apps for IMproving FITness and Increasing Physical Activity Among Young People: The AIMFIT Pragmatic Randomized Controlled Trial. J Med Internet Res. 2015;17.

Donnelly JE, Greene JL, Gibson CA, Smith BK, Washburn RA, Sullivan DK, et al. Physical Activity Across the Curriculum (PAAC): A randomized controlled trial to promote physical activity and diminish overweight and obesity in elementary school children. Prev Med (Baltim). 2009;49:336–41.

Dudley DA, Okely AD, Pearson P, Peat J. Engaging adolescent girls from linguistically diverse and low income backgrounds in school sport: A pilot randomised controlled trial. J Sci Med Sport. 2010;13:217–24. doi:10.1016/j.jsams.2009.04.008.

Duncan S, McPhee JC, Schluter PJ, Zinn C, Smith R, Schofield G. Efficacy of a compulsory homework programme for increasing physical activity and healthy eating in children: The Healthy Homework pilot study. Int J Behav Nutr Phys Act. 2011;8:no pagination-no pagination. doi:10.1186/1479-5868-8-127.

Eather N, Morgan PJ, Lubans DR. Improving the fitness and physical activity levels of primary school children: results of the Fit-4-Fun group randomized controlled trial. Prev Med (Baltim). 2013;56:12–9.

Eather N, Morgan PJ, Lubans DR. Feasibility and preliminary efficacy of the Fit4Fun intervention for improving physical fitness in a sample of primary school children: a pilot study. Phys Educ Sport Pedagog. 2013;18:389–411.

Engelen L, Bundy AC, Naughton G, Simpson JM, Bauman A, Ragen J, et al. Increasing physical activity in young primary school children—It’s child’s play: A cluster randomised controlled trial. Prev Med An Int J Devoted to Pract Theory. 2013;56:319–25.

Erwin HE, Beighle A, Morgan CF, Noland M. Effect of a low-cost, teacher-directed classroom intervention on elementary students’ physical activity. J Sch Health. 2011;81:455–61.

Ezendam NPM, Brug J, Oenema A, JJ R, I A, PM G, et al. Evaluation of the Web-Based Computer-Tailored FATaintPHAT Intervention to Promote Energy Balance Among Adolescents. Arch Pediatr Adolesc Med. 2012;166:248.

Eyre ELJ, Cox VM, Birch SL, Duncan MJ. An integrated curriculum approach to increasing habitual physical activity in deprived South Asian children. Eur J Sport Sci. 2016;16:381–90.

Fairclough SJ, Hackett AF, Davies IG, Gobbi R, Mackintosh KA, Warburton GL, et al. Promoting healthy weight in primary school children through physical activity and nutrition education: a pragmatic evaluation of the CHANGE! randomised intervention study. BMC Public Health. 2013;13:626.

Finkelstein EA, Tan Y-C, Malhotra R, Lee C-F, Goh S-S, Saw S-M. A cluster randomized controlled trial of an incentive-based outdoor physical activity program. J Pediatr. 2013;163:167–72.e1.

Ford P a., Perkins G, Swaine I. Effects of a 15-week accumulated brisk walking programme on the body composition of primary school children. J Sports Sci. 2012;414 August 2015:1–9.

Gentile DA, Welk G, Eisenmann JC, Reimer RA, Walsh DA, Russell DW, et al. Evaluation of a multiple ecological level child obesity prevention program: Switch what you Do, View, and Chew. BMC Med. 2009;7:49.

Gerards SMPL, Dagnelie PC, Gubbels JS, van Buuren S, Hamers FJM, Jansen MWJ, et al. The effectiveness of lifestyle triple P in the Netherlands: a randomized controlled trial. PLoS One. 2015;10:e0122240–e0122240.

Going S, Thompson J, Cano S, Stewart D, Stone E, Harnack L, et al. The effects of the Pathways Obesity Prevention Program on physical activity in American Indian children. Prev Med (Baltim). 2003;37 SUPPL. 1:S62-9.

Goran MI, Reynolds K. Interactive Multimedia for Promoting Physical Activity (IMPACT) in Children. Obes Res. 2005;13:762–71.

Gorely T, Morris JG, Musson H, Brown S, Nevill A, Nevill ME. Physical activity and body composition outcomes of the GreatFun2Run intervention at 20 month follow-up. Int J Behav Nutr Phys Act. 2011;8:74.

Gorely T, Nevill ME, Morris JG, Stensel DJ, Nevill A. Effect of a school-based intervention to promote healthy lifestyles in 7–11 year old children. Int J Behav Nutr Phys Act. 2009;6.

Gortmaker SL, Lee RM, Mozaffarian RS, Sobol AM, Nelson TF, Roth BA, et al. Effect of an after-school intervention on increases in children’s physical activity. Med Sci Sports Exerc. 2012;44:450–7.

Graves  Ridgers  Atkinson, G., Stratton, G., L N. The Effect of Active Video Gaming on Children’s Physical Activity , Behavior Preferences and Body Composition. Pediatr Excercise Sci. 2010;22 April 2016:535–46.

Grydeland M, Bergh IH, Bjelland M, Lien N, Andersen LF, Ommundsen Y, et al. Intervention effects on physical activity: The HEIA study - a cluster randomized controlled trial. Int J Behav Nutr Phys Act. 2013;10:17.

Ha AS, Burnett A, Sum R, Medic N, Ng JYY. Outcomes of the Rope Skipping “STAR” Programme for Schoolchildren. J Hum Kinet. 2015;45:233–40.

Haerens L, De Bourdeaudhuij I, Maes L, Cardon G, Deforche B. School-Based Randomized Controlled Trial of a Physical Activity Intervention among Adolescents. J Adolesc Heal. 2007;40:258–65.

Haerens L, Deforche B, Maes L, Cardon G, Stevens V, Bourdeaudhuij I De. Evaluation of a 2-year physical activity and healthy eating intervention in middle school children. Health Educ Res. 2006;21:911–21.

Hands B, Larkin D, Rose E, Parker H, Smith A. Can Young Children Make Active Choices? Outcomes of a Feasibility Trial in Seven-Year-Old Children. Early Child Dev Care. 2011;181:625–37.

Harder-lauridsen NM, Birk NM, Ried-larsen M, Juul A, Andersen LB. A randomized controlled trial on a multicomponent intervention for overweight school-aged children - Copenhagen, Denmark. BMC Pediatr. 2014;273:1–14.

Hardman CA, Horne PJ, Lowe CF. A home-based intervention to increase physical activity in girls: The fit “n” fun dudes program. J Exerc Sci Fit. 2009;7:1–8.

Hardman CA, Horne PJ, Lowe CF. Effects of rewards, peer-modelling and pedometer targets on children’s physical activity: A school-based intervention study. Psychol Heal. 2011;26:3–21.

Heelan  Abbey  Donnelly  Mayo, M., Welk, G., K, B J. Evaluation of a Walking School Bus for Promoting Physical Activity in Youth. J Phys Act Heal. 2009;6:560–7.

Herrick H, Thompson H, Kinder J, Madsen KA. Use of SPARK to Promote After-School Physical Activity. J Sch Health. 2012;82:457–61.

Jago R, Sebire SJ, Turner KM, Bentley GF, Goodred JK, Fox KR, et al. Feasibility trial evaluation of a physical activity and screen-viewing course for parents of 6 to 8 year-old children: Teamplay. Int J Behav Nutr Phys Act. 2013;10.

Jago R, Baranowski T, Baranowski JC, Thompson D, Cullen KW, Watson K, et al. Fit for Life Boy Scout badge: Outcome evaluation of a troop and Internet intervention. Prev Med An Int J Devoted to Pract Theory. 2006;42:181–7.

Jago R, Edwards MJ, Sebire SJ, Tomkinson K, Bird EL, Banfield K, et al. Effect and cost of an after-school dance programme on the physical activity of 11-12 year old girls: The Bristol Girls Dance Project, a school-based cluster randomised controlled trial. Int J Behav Nutr Phys Act. 2015;12:128.

Jago R, Sebire SJ, Cooper AR, Haase AM, Powell J, Davis L, et al. Bristol Girls Dance Project Feasibility Trial: Outcome and process evaluation results. Int J Behav Nutr Phys Act. 2012;9:83.

Jago R, Sebire SJ, Davies B, Wood L, Edwards MJ, Banfield K, et al. Randomised feasibility trial of a teaching assistant led extracurricular physical activity intervention for 9 to 11 year olds: Action 3:30. Int J Behav Nutr Phys Act. 2014;11.

Kipping RR, Howe LD, Jago R, Campbell R, Wells S, Chittleborough CR, et al. Effect of intervention aimed at increasing physical activity, reducing sedentary behaviour, and increasing fruit and vegetable consumption in children: Active for Life Year 5 (AFLY5) school based cluster randomised controlled trial. BMJ Br Med J. 2014;348.

Klesges R, Obarzanek E, Kumanyika S, Murray D, Klesges L, Relyea G, et al. The Memphis Girls’ health Enrichment Multi-site Studies (GEMS). Arch Pediatr Adolesc Med. 2010;164:1007–14.

Kriemler S, Zahner L, Schindler C, Meyer U, Hartmann T, Hebestreit H, et al. Effect of school based physical activity programme (KISS) on fitness and adiposity in primary schoolchildren: cluster randomised controlled trial Kriemler S. BMJ. 2010;340:c785–c785.

Laukkanen A, Juhani Pesola A, Heikkinen R, Kaarina Sääkslahti A, Finni T. Family-based cluster randomized controlled trial enhancing physical activity and motor competence in 4-7-year-old children. PLoS One. 2015;10.

Lee L, Kuo Y, Fanaw D, Perng S, Juang I. The effect of an intervention combining self efficacy theory and pedometers on promoting physical activity among adolescents. J Clin Nurs. 2012;21:914–22.

Lubans DR, Morgan PJ, Callister R, Collins CE, Plotnikoff RC. Exploring the mechanisms of physical activity and dietary behavior change in the program x intervention for adolescents. J Adolesc Health. 2010;47:83–91.

Lubans DR, Morgan PJ, Okely AD, Dewar D, Collins CE, Batterham M, et al. Preventing obesity among adolescent girls: One-year outcomes of the nutrition and enjoyable activity for teen girls (NEAT Girls) cluster randomized controlled trial. Arch Pediatr Adolesc Med. 2012;166:821–7.

Lubans DR, Morgan PJ, Aguiar EJ, Callister R. Randomized controlled trial of the Physical Activity Leaders (PALs) program for adolescent boys from disadvantaged secondary schools. Prev Med (Baltim). 2011;52:239–46.

Lubans DR, Morgan PJ, Callister R, P.J. M. Potential moderators and mediators of intervention effects in an obesity prevention program for adolescent boys from disadvantaged schools. J Sci Med Sport. 2012;15:519–25.

Lubans DR, Morgan PJ, Callister R, Collins CE. Effects of integrating pedometers, parental materials, and e-mail support within an extracurricular school sport intervention. J Adolesc Heal. 2009;44:176–83.

Lubans D, Morgan P. Evaluation of an extra-curricular school sport programme promoting lifestyle and lifetime activity for adolescents. J Sports Sci. 2008;26:519–29.

MacConnie SE, Gilliam TB, Geenen DL, Pels AE. Daily physical activity patterns of prepubertal children involved in a vigorous exercise program. Int J Sports Med. 1982;3:202–7.

Madsen K, Linchey J, Gerstein D, Ross M, Myers E, Brown K, et al. Energy Balance 4 Kids with Play: Results from a Two-Year Cluster-Randomized Trial. Child Obes. 2015;11:375–83.

Madsen K, Thompson H, Adkins A, Crawford Y. School-Community Partnerships: A Cluster-Randomized Trial of an After-School Soccer Program. JAMA Pediatr. 2013;167:321.

Magnusson KT, Sigurgeirsson I, Sveinsson T, Johannsson E. Assessment of a two-year school-based physical activity intervention among 7–9–year-old children. Int J Behav Nutr Phys Act. 2011;8:138.

Maloney AE, Bethea TC, Kelsey KS, Marks JT, Paez S, Rosenberg AM, et al. A pilot of a video game (DDR) to promote physical activity and decrease sedentary screen time. Obesity. 2008;16:2074–80.

Marcus C, Nyberg G, Nordenfelt A, Karpmyr M, Kowalski J, Ekelund U. A 4-year, cluster-randomized, controlled childhood obesity prevention study: STOPP. Int J Obes. 2009;33:408–17.

McManus AM, Masters RSW, Laukkanen RMT, Yu CCW, Sit CHP, Ling FCM. Using heart-rate feedback to increase physical activity in children. Prev Med (Baltim). 2008;47:402–8.

McMinn D, Rowe DA, Murtagh S, Nelson NM. The effect of a school-based active commuting intervention on children’s commuting physical activity and daily physical activity. Prev Med (Baltim). 2012;54:316–8.

Meinhardt U, Witassek F, Petrò R, Fritz C, Eiholzer U. Strength training and physical activity in boys: a randomized trial. Pediatrics. 2013;132:1105–11.

Mendoza J a., Watson K, Baranowski T, Nicklas T a., Uscanga DK, Hanfling MJ. The Walking School Bus and Children’s Physical Activity: A Pilot Cluster Randomized Controlled Trial. Pediatrics. 2011;128.

Meyer U, Schindler C, Zahner L, Ernst D, Hebestreit H, van Mechelen W, et al. Long-term effect of a school-based physical activity program (KISS) on fitness and adiposity in children: A cluster-randomized controlled trial Meyer U. PLoS One. 2014;9.

Morgan PJ, Lubans DR, Callister R, Okely AD, Burrows TL, Fletcher R, et al. The Healthy Dads, Healthy Kids randomized controlled trial: Efficacy of a healthy lifestyle program for overweight fathers and their children. Int J Obes (Lond). 2011;35:436–47.

Mhurchu CN, Maddison R, Jiang Y, Jull A, Prapavessis H, Rodgers A. Couch potatoes to jumping beans: A pilot study of the effect of active video games on physical activity in children. Int J Behav Nutr Phys Act. 2008;5:8.

Morgan PJ, Collins CE, Plotnikoff RC, Callister R, Burrows T, Fletcher R, et al. The “Healthy Dads, Healthy Kids” community randomized controlled trial: A community-based healthy lifestyle program for fathers and their children. Prev Med An Int J Devoted to Pract Theory. 2014;61:90–9.

Morris JG, Gorely T, Sedgwick MJ, Nevill A, Nevill ME, J.G. M, et al. Effect of the Great Activity Programme on healthy lifestyle behaviours in 7-11 year olds. J Sports Sci. 2013;31:1280–93.

Morrison R, Reilly JJ, Penpraze V, Westgarth C, Ward DS, Mutrie N, et al. Children, parents and pets exercising together (CPET): exploratory randomised controlled trial. BMC Public Health. 2013;13:1096.

Naylor P-J, Macdonald HM, Warburton DER, Reed KE, McKay HA, H.M. M, et al. An active school model to promote physical activity in elementary schools: Action schools! BC Naylor P.-J. Br J Sports Med. 2008;42:338–43.

Nyberg G, Norman A, Sundblom E, Zeebari Z, Elinder LS, G. N, et al. Effectiveness of a universal parental support programme to promote health behaviours and prevent overweight and obesity in 6-year-old children in disadvantaged areas, the Healthy School Start Study II, a cluster-randomised controlled trial. Int J Behav Nutr Phys Act. 2016;13:4.

Nyberg G, Sundblom E, Norman Å, Bohman B, Hagberg J, Elinder LS, et al. Effectiveness of a Universal Parental Support Programme to Promote Healthy Dietary Habits and Physical Activity and to Prevent Overweight and Obesity in 6-Year-Old Children: The Healthy School Start Study, a Cluster-Randomised Controlled Trial. PLoS One. 2015;10:e0116876–e0116876.

O’Brien W, Issartel J, Belton S. Evidence for the Efficacy of the Youth-Physical Activity towards Health ( Y-PATH ) Intervention. Adv Phys Educ. 2013;3:145–53.

Olvera N, Bush JA, Sharma S V, Knox BB, Scherer RL, Butte NF. BOUNCE: A community-based mother-daughter healthy lifestyle intervention for low-income Latino families. Obesity. 2010;18 Suppl 1:S102–4.

Pangrazi RP, Beighle A, Vehige T, Vack C, R.P. P, A. B, et al. Impact of Promoting Lifestyle Activity for Youth (PLAY) on children’s physical activity. J Sch Health. 2003;73:317–21.

Pardo BM, Bengoechea EG, Julián Clemente JA, Lanaspa EG. Empowering adolescents to be physically active: Three-year results of the Sigue la Huella intervention. Prev Med (Baltim). 2014;66:6–11.

Patrick K, KJ C, GJ N, al et. Randomized controlled trial of a primary care and home-based intervention for physical activity and nutrition behaviors: Pace+ for adolescents. Arch Pediatr Adolesc Med. 2006;160:128–36.

Prochaska JJ, Sallis JF. A Randomized Controlled Trial of Single Versus Multiple Health Behavior Change: Promoting Physical Activity and Nutrition Among Adolescents. Heal Psychol. 2004;23:314–8.

Puder JJ, Marques-Vidal P, Schindler C, Zahner L, Niederer I, Bürgi F, et al. Effect of multidimensional lifestyle intervention on fitness and adiposity in predominantly migrant preschool children (Ballabeina): cluster randomised controlled trial. BMJ. 2011;343:d6195–d6195.

Reza S, Tahir WM, Zakaria W, Agency MN, Agency N. Impact of Social-Ecological Intervention on Physical Activity Knowledge and Behaviors of Rural Students. J Phys Act Heal. 2014.

Reznik M, Wylie-Rosett J, Kim M, Ozuah PO. A classroom-based physical activity intervention for urban kindergarten and first-grade students: A feasibility study. Child Obes. 2015;11:314–24.

Riley N, Lubans DR, Morgan PJ, Young M. Outcomes and process evaluation of a programme integrating physical activity into the primary school mathematics curriculum: The EASY Minds pilot randomised controlled trial. J Sci Med Sport. 2015;18:656–61.

Robbins LB, Pfeiffer KA, Maier KS, Lo Y-J, Wesolek SM. Pilot Intervention to Increase Physical Activity Among Sedentary Urban Middle School Girls: A Two-Group Pretest-Posttest Quasi-Experimental Design. J Sch Nurs. 2012;28:302–15.

Roemmich JN, Gurgol CM, Epstein LH. Open-Loop Feedback Increases Physical Activity of Youth. Med Sci Sport Exerc. 2004;36:668–73.

Roemmich JN, Lobarinas CL, Barkley JE, White TM, Paluch R, Epstein LH. Use of an open-loop system to increase physical activity. Pediatr Exerc Sci. 2012;24:384–98.

Salmon J, Ball K, Hume C, Booth M, Crawford D. Outcomes of a group-randomized trial to prevent excess weight gain, reduce screen behaviours and promote physical activity in 10-year-old children: switch-play. Int J Obes (Lond). 2008;32:601–12.

Schofield L, Mummery WK, Schofield G. Effects of a controlled pedometer-intervention trial for low-active adolescent girls. Med Sci Sports Exerc. 2005;37:1414–20.

Serra-Paya N, Ensenyat A, Castro-Vinuales I, Real J, Sinfreu-Bergues X, Zapata A, et al. Effectiveness of a Multi-Component Intervention for Overweight and Obese Children (Nereu Program): A Randomized Controlled Trial. PLoS One. 2015;10:e0144502–e0144502.

Shore SM, Sachs ML, DuCette JP, Libonati JR. Step-Count Promotion Through a School-Based Intervention. Clin Nurs Res. 2014;23:402–20.

Sigmund E, El Ansari W, Sigmundová D. Does school-based physical activity decrease overweight and obesity in children aged 6-9 years? A two-year non-randomized longitudinal intervention study in the Czech Republic. BMC Public Health. 2012;12:570.

Smith JJ, Morgan PJ, Plotnikoff RC, Dally KA, Salmon J, Okely AD, et al. Smart-phone obesity prevention trial for adolescent boys in low-income communities: The ATLAS RCT. Pediatrics. 2014;134:e723–31.

Story M, Sherwood NE, Himes JH, Davis M, Jacobs DR, Cartwright Y, et al. An After-school obesity prevention program for africian-americian girls: The Minnesota GEMS Pilot Study. Ethn Dis. 2003;13.

Straker LM, Abbott RA, Smith AJ. To remove or to replace traditional electronic games? A crossover randomised controlled trial on the impact of removing or replacing home access to electronic games on physical activity and sedentary behaviour in children aged 10-12 years. BMJ Open. 2013;3.

Suchert V, Isensee B, Sargent J, Weisser B, Hanewinkel R, Group lauft. S. Prospective effects of pedometer use and class competitions on physical activity in youth: A cluster-randomized controlled trial. Prev Med (Baltim). 2015;81:399–404.

Sutherland R, Campbell E, Lubans DR, Morgan PJ, Okely AD, Nathan N, et al. “Physical Activity 4 Everyone” school-based intervention to prevent decline in adolescent physical activity levels: 12 month (mid-intervention) report on a cluster randomised trial. British Journal of Sports Medicine. 2015.

Sutherland RL, Campbell EM, Lubans DR, Morgan PJ, Nathan NK, Wolfenden L, et al. The Physical Activity 4 Everyone Cluster Randomized Trial: 2-year Outcomes of a School Physical Activity Intervention Among Adolescents. Am J Prev Med. 2016;:1–11.

Taylor RW, Mcauley KA, Barbezat W, Strong A, Williams SM, Mann JI. APPLE Project : 2-y findings of a community-based obesity prevention program in primary school – age children. Am J Clin Nutr. 2007.

Taylor RW, Mcauley KA, Williams SM, Barbezat W, Nielsen G, Mann JI, et al. Reducing weight gain in children through enhancing physical activity and nutrition: the APPLE project. Int J Pediatr Obes. 2006;1:146–52.

Toftager M, Christiansen LB, Ersbøll AK, Kristensen PL, Due P, Troelsen J. Intervention Effects on Adolescent Physical Activity in the Multicomponent SPACE Study: A Cluster Randomized Controlled Trial. PLoS One. 2014;9:e99369–e99369.

Tymms PB, Curtis SE, Routen AC, Thomson KH, Bolden DS, Bock S, et al. Clustered randomised controlled trial of two education interventions designed to increase physical activity and well-being of secondary school students: the MOVE Project. BMJ Open. 2016;6:e009318–e009318.

Verbestel V, De Henauw S, Barba G, Eiben G, Gallois K, Hadjigeorgiou C, et al. Effectiveness of the IDEFICS intervention on objectively measured physical activity and sedentary time in European children. Obes Rev. 2015;16 Suppl 2:57–67.

Verloigne M, Bere E, Van Lippevelde W, Maes L, Lien N, Vik FN, et al. The effect of the UP4FUN pilot intervention on objectively measured sedentary time and physical activity in 10-12 year old children in Belgium: the ENERGY-project. BMC Public Health. 2012;12:805.

Verstraete SJM, Cardon GM, De Clercq DLR, De Bourdeaudhuij IMM. A comprehensive physical activity promotion programme at elementary school: The effects on physical activity, physical fitness and psychosocial correlates of physical activity. Public Health Nutr. 2007;10:477–84.

Wells NM, Myers BM, Henderson Jr. CR. School gardens and physical activity: A randomized controlled trial of low-income elementary schools. Prev Med (Baltim). 2014;69, Supple:S27–33.

Wilson DK, Evans AE, Williams J, Mixon G, Sirard JR, Pate R, et al. A preliminary test of a student-centered intervention on increasing physical activity in underserved adolescents. Ann Behav Med. 2005;30:119–24.

Wilson DK, Friend R, Teasley N, Green S, Reaves IL, Sica DA. Motivational versus social cognitive interventions for promoting fruit and vegetable intake and physical activity in African American adolescents. Ann Behav Med. 2002;24:310–9.

Wilson DK, Van Horn ML, Kitzman-Ulrich H, Saunders R, Pate R, Lawman HG, et al. Results of the “ Active by Choice Today ” (ACT) Randomized Trial for Increasing Physical Activity in Low-Income and Minority Adolescents. Health Psychol. 2011;30:463–71.

**File S4: Associated publications:**

Adkins S, Sherwood NE, Story M, Davis M. Physical activity among African-American girls: the role of parents and the home environment. Obes Res. 2004;12 Suppl September:38S–45S.

Anderson EL, Howe LD, Kipping RR, Campbell R, Jago R, Noble SM, et al. Long-term effects of the Active for Life Year 5 (AFLY5) school-based cluster-randomised controlled trial. BMJ Open. 2016;6:1–11.

Andrade S, Verloigne M, Cardon G, Kolsteren P, Ochoa-Avilés A, Verstraeten R, et al. School-based intervention on healthy behaviour among Ecuadorian adolescents: effect of a cluster-randomized controlled trial on screen-time. BMC Public Health. 2015;15:942.

Bammann K, Gwozdz W, Lanfer A, Barba G, De Henauw S, Eiben G, et al. Socioeconomic factors and childhood overweight in europe: Results from the multi-centre IDEFICS study. Pediatr Obes. 2013;8:1–12.

Baranowski T, Lytle L. Should the IDEFICS outcomes have been expected? Obes Rev. 2015;16 December:162–72.

Beckvid Henriksson G, Franzén S, Elinder LS, Nyberg G. Low socioeconomic status associated with unhealthy weight in six-year-old Swedish children despite higher levels of physical activity. Acta Paediatr. 2016;:n/a-n/a.

Belton S, O’ Brien W, Meegan S, Woods C, Issartel J. Youth-Physical Activity Towards Health: evidence and background to the development of the Y-PATH physical activity intervention for adolescents. BMC Public Health. 2014;14:122.

Bergh IH, van Stralen MM, Grydeland M, Bjelland M, Lien N, Andersen LF, et al. Exploring mediators of accelerometer assessed physical activity in young adolescents in the health in adolescents study -- a group randomized controlled trial. BMC Public Health. 2012;12:814.

Bergh IH, Bjelland M, Grydeland M, Lien N, Andersen LF, Klepp K-I, et al. Mid-way and post-intervention effects on potential determinants of physical activity and sedentary behavior, results of the HEIA study - a multicomponent school-based randomized trial. Int J Behav Nutr Phys Act. 2012;9:63.

Bergh IH, van Stralen MM, Bjelland M, Grydeland M, Lien N, Klepp KI, et al. Post-intervention effects on screen behaviours and mediating effect of parental regulation: the HEalth In Adolescents study--a multi-component school-based randomized controlled trial. BMC Public Health. 2014;14:200.

Bjelland M, Soenens B, Bere E, Kovács É, Lien N, Maes L, et al. Associations between parental rules, style of communication and children’s screen time. BMC Public Health. 2015;15:1–13.

Boddy LM, Knowles ZR, Davies IG, Warburton GL, Mackintosh KA, Houghton L, et al. Using formative research to develop the healthy eating component of the CHANGE! school-based curriculum intervention. BMC Public Health. 2012;12:710.

Breslin G, Brennan D. A Healthy Lifestyle Intervention Delivered by Aspiring Physical Education Teachers to Children from Social Disadvantage: Study Protocol and Preliminary Findings. Child Care Pract. 2012;18 May 2015:207–25.

Brien WO, Belton S, Issartel J. Promoting physical literacy in Irish adolescent youth: The Youth-Physical Activity Towards Health (Y-PATH) intervention. MOJ Public Heal. 2015;2:1–6.

Bundy AC, Naughton G, Tranter P, Wyver S, Baur L, Schiller W, et al. The Sydney playground project: popping the bubblewrap--unleashing the power of play: a cluster randomized controlled trial of a primary school playground-based intervention aiming to increase children’s physical activity and social skills. BMC Public Health. 2011;11:680.

Bürgi F, Niederer I, Schindler C, Bodenmann P, Marques-Vidal P, Kriemler S, et al. Effect of a lifestyle intervention on adiposity and fitness in socially disadvantaged subgroups of preschoolers: A cluster-randomized trial (Ballabeina). Prev Med (Baltim). 2012;54:335–40.

Chen J, Cooper B. The Efficacy of the Web ABC Program on Improving and Quality of Life in Chinese American Adolescents. ICAN Infant, Child, Adolesc Nutr. 2012;4.

Chen J-L, Weiss SJ, Heyman MB, Cooper B, Lustig RH. ABC program for improving coping and quality of life in Chinese-American children. Nurs Res. 2010;59:270–9.

Davis CE, Hunsberger S, Murray DM, Fabsitz RR, Himes JH, Stephenson LK, et al. Design and statistical analysis for the Pathways study 1–3. Am J Clin Nutr. 1999;69:760–3.

De Henauw S, Verbestel V, Mårild S, Barba G, Bammann K, Eiben G, et al. The IDEFICS community-oriented intervention programme: a new model for childhood obesity prevention in Europe? Int J Obes (Lond). 2011;35 Suppl 1:S16–23.

de Bourdeaudhuij I, Verbestel V, De Henauw S, Maes L, Huybrechts I, Mårild S, et al. Behavioural effects of a community-oriented setting-based intervention for prevention of childhood obesity in eight European countries. Main results from the IDEFICS study. Obes Rev. 2015;16 December:30–40.

Donnelly JE, Greene JL, Gibson CA, Sullivan DK, Hansen DM, Hillman CH, et al. Physical activity and academic achievement across the curriculum (A + PAAC): rationale and design of a 3-year, cluster-randomized trial. BMC Public Health. 2013;13:307.

Drummy C, Murtagh EM, McKee DP, Breslin G, Davison GW, Murphy MH. The effect of a classroom activity break on physical activity levels and adiposity in primary school children. J Paediatr Child Health. 2016;52:745–9.

DuBose KD, Mayo MS, Gibson CA, Green JL, Hill JO, Jacobsen DJ, et al. Physical activity across the curriculum (PAAC): Rationale and design. Contemp Clin Trials. 2008;29:83–93.

Eather N, Morgan PJ, Lubans DR. Social support from teachers mediates physical activity behavior change in children participating in the Fit-4-Fun intervention. Int J Behav Nutr Phys Act. 2013;10:68.

Edwards MJ, May T, Kesten JM, Banfield K, Bird EL, Powell JE, et al. Lessons learnt from the Bristol Girls Dance Project cluster RCT: implications for designing and implementing after-school physical activity interventions. BMJ Open. 2016;6:e010036.

Eisenmann JC, Gentile DA, Welk GJ, Callahan R, Strickland S, Walsh M, et al. SWITCH: rationale, design, and implementation of a community, school, and family-based intervention to modify behaviors related to childhood obesity. BMC Public Health. 2008;8:223.

Ezendam NPM, Noordegraaf VSA, Kroeze W, Brug J, Oenema A. Process evaluation of FATaintPHAT, a computer-tailored intervention to prevent excessive weight gain among Dutch adolescents. Health Promot Int. 2013;28:26–35.

Ezendam NPM, Burg J, Borsboom G, van Empelen P, Oenema A. 15. Differential effects of the computer-tailored FATaintPHAT programme on dietary behaviours according to sociodemographic, cognitive and home environmental factors. Public Health Nutr. 2014;17:431–9.

Finni T, Saakslahti A, Laukkanen A, Pesola A, Sipila S, Sääkslahti A, et al. A family based tailored counselling to increase non-exercise physical activity in adults with a sedentary job and physical activity in their young children: design and methods of a year-long randomized controlled trial. BMC Public Health. 2011;11:944.

Gibson C, Smith B, DuBose K, Greene L, Bailey B, Williams S, et al. Physical activity across the curriculum: year one process evaluation results. Int J Behav Nutr Phys Act. 2008;5:1–11.

Haerens L, Cerin E, Maes L, Cardon G, Deforche B, De Bourdeaudhuij I. Explaining the effect of a 1-year intervention promoting physical activity in middle schools: a mediation analysis. Public Health Nutr. 2008;11:501–12.

Haerens L, Deforche B, Maes L, Stevens V, Cardon G, De Bourdeaudhuij I. Body mass effects of a physical activity and healthy food intervention in middle schools. Obesity (Silver Spring). 2006;14 May:847–54.

Hartmann T, Zahner L, Pühse U, Schneider S, Puder JJ, Kriemler S. Physical activity, bodyweight, health and fear of negative evaluation in primary school children. Scand J Med Sci Sport. 2010;20:27–34.

Hartmann T, Zahner L, Pühse U, Puder JJ, Kriemler S. Effects of a school-based physical activity program on physical and psychosocial quality of life in elementary school children: a cluster-randomized trial. Pediatr Exerc Sci. 2010;22:511–22.

Hasselstrøm HA, Karlsson MK, Hansen SE, Grønfeldt V, Froberg K, Andersen LB. A 3-Year Physical Activity Intervention Program Increases the Gain in Bone Mineral and Bone Width in Prepubertal Girls but not Boys: The Prospective Copenhagen School Child Interventions Study (CoSCIS). Calcif Tissue Int. 2008;83:243–50.

Hollis JL, Sutherland R, Campbell L, Morgan PJ, Lubans DR, Nathan N, et al. Effects of a “school-based” physical activity intervention on adiposity in adolescents from economically disadvantaged communities: secondary outcomes of the “Physical Activity 4 Everyone” RCT. Int J Obes. 2016;40 April:1–8.

Honas JJ, Washburn RA, Smith BK, Greene JL, Donnelly JE. Energy expenditure of the physical activity across the curriculum intervention. Med Sci Sports Exerc. 2008;40:1501–5.

Jago R, Rawlins E, Kipping RR, Wells S, Chittleborough C, Peters TJ, et al. Lessons learned from the AFLY5 RCT process evaluation: implications for the design of physical activity and nutrition interventions in schools. BMC Public Health. 2015;15:946.

Jago R, Sebire SJ, Bentley GF, Turner KM, Goodred JK, Fox KR, et al. Process evaluation of the Teamplay parenting intervention pilot: implications for recruitment, retention and course refinement. BMC Public Health. 2013;13:1102.

Jago R, Sebire SJ, Davies B, Wood L, Banfield K, Edwards MJ, et al. Increasing children’s physical activity through a teaching-assistant led extracurricular intervention: process evaluation of the action 3:30 randomised feasibility trial. BMC Public Health. 2015;15:156.

Laukkanen A, Pesola A, Havu M, S????kslahti A, Finni T. Relationship between habitual physical activity and gross motor skills is multifaceted in 5- to 8-year-old children. Scand J Med Sci Sport. 2014;24:102–10.

Lawlor DA, Howe LD, Anderson EL, Kipping RR, Campbell R, Wells S, et al. The Active for Life Year 5 (AFLY5) school-based cluster randomised controlled trial: Effect on potential mediators. BMC Public Health. 2016;16:1–21.

Lawlor DA, Jago R, Noble SM, Chittleborough CR, Campbell R, Mytton J, et al. The Active for Life Year 5 (AFLY5) school based cluster randomised controlled trial: Study protocol for a randomized controlled trial. Trials. 2011;12:no pagination-no pagination.

Lawman H, Wilson D, Van Horn L, Resnicow K, Kitzman-Ulrich H. The Realationship between Psychosocial Correlates and Physical Activity in Underserved Adolescent Boys and Girls in the ACT Trial. J Phys (main title). 2011;8:253–61.

Lehne G, Bolte G. Equity impact of interventions to promote physical activity in older adults: protocol for a systematic review. Syst Rev. 2016;5:17.

Lehne G, Bolte G. Impact of universal interventions on social inequalities in physical activity among older adults: an equity-focused systematic review. Int J Behav Nutr Phys Act. 2017;14:20.

Lissner L, de Bourdeaudhuij I, Konstabel K, Mårild S, Mehlig K, Molnár D, et al. Differential outcome of the IDEFICS intervention in overweight versus non-overweight children: Did we achieve “primary” or “secondary” prevention? Obes Rev. 2015;16 December:119–26.

Lubans DR, Morgan PJ, Weaver K, Callister R, Dewar DL, Costigan SA, et al. Rationale and study protocol for the supporting children’s outcomes using rewards, exercise and skills (SCORES) group randomized controlled trial: a physical activity and fundamental movement skills intervention for primary schools in low-income communiti. BMC Public Health. 2012;12:427.

Lubans DR, Morgan PJ, Dewar D, Collins CE, Plotnikoff RC, Okely AD, et al. The Nutrition and Enjoyable Activity for Teen Girls (NEAT girls) randomized controlled trial for adolescent girls from disadvantaged secondary schools: rationale, study protocol, and baseline results. BMC Public Health. 2010;10 October:652.

Magnusson KT, Hrafnkelsson H, Sigurgeirsson I, Johannsson E, Sveinsson T. Limited effects of a 2-year school-based physical activity intervention on body composition and cardiorespiratory fitness in 7-year-old children. Health Educ Res. 2012;27:484–94.

Martin R, Murtagh E. Active Classrooms: A Cluster Randomised Controlled Trial Evaluating the Effects of a Movement Integration Intervention on the Physical Activity Levels of Primary School Children Authors: Int J Sport Nutr Exerc Metab. 2016;32:1–44.

McAuley K a, Taylor RW, Farmer VL, Hansen P, Williams SM, Booker CS, et al. Economic evaluation of a community-based obesity prevention program in children: the APPLE project. Obesity (Silver Spring). 2010;18:131–6.

Michels N, Susi K, Marques-Vidal PM, Nydegger A, Puder JJ. Psychosocial Quality-of-Life, Lifestyle and Adiposity: A Longitudinal Study in Pre-schoolers (Ballabeina Study). Int J Behav Med. 2016;:1–10.

Murillo Pardo B, García Bengoechea E, Julián Clemente JA, Generelo Lanaspa E. Motivational Outcomes and Predictors of Moderate-to-Vigorous Physical Activity and Sedentary Time for Adolescents in the Sigue La Huella Intervention. Int J Behav Med. 2016;23:135–42.

Niederer I, Bürgi F, Ebenegger V, Marques-Vidal P, Schindler C, Nydegger A, et al. Effects of a lifestyle intervention on adiposity and fitness in overweight or low fit preschoolers (Ballabeina). Obesity. 2013;21:287–93.

Niederer I, Kriemler S, Zahner L, Bürgi F, Ebenegger V, Hartmann T, et al. Influence of a lifestyle intervention in preschool children on physiological and psychological parameters (Ballabeina): study design of a cluster randomized controlled trial. BMC Public Health. 2009;9:94.

Norman Å, Nyberg G, Elinder LS, Berlin A. One size does not fit all-qualitative process evaluation of the Healthy School Start parental support programme to prevent overweight and obesity among children in disadvantaged areas in Sweden. BMC Public Health. 2016;16:37.

Nyberg G, Sundblom E, Norman Å, Bohman B, Hagberg J, Elinder LS. Effectiveness of a universal parental support programme to promote healthy dietary habits and physical activity and to prevent overweight and obesity in 6-year-old children: The healthy school start study, a cluster-randomised controlled trial. PLoS One. 2015;10:1–14.

Nyberg G, Sundblom E, Norman A, Elinder LS. A healthy school start - parental support to promote healthy dietary habits and physical activity in children: design and evaluation of a cluster-randomised intervention. BMC Public Health. 2011;11:185.

Olvera NN, Knox B, Scherer R, Maldonado G, Sharma S V., Alastuey L, et al. A Healthy Lifestyle Program for Latino Daughters and Mothers. Am J Heal Educ. 2008;39:283–95. 10599052.

Olvera N, Graham M, McLeod J, Kellam SF, Butte NF. Promoting moderate-vigorous physical activity in overweight minority girls. Int J Pediatr. 2010.

Paez S, Maloney A, Kelsey K, Wiesen C, Rosenberg A. Parental and environmental factors associated with physical activity among children participating in an active video game. Pediatr Phys Ther. 2009;21:245–53.

Pardo BM, Bengoechea EG, Solana AA, Clemente JAJ, García-González L, Martín-Albo J, et al. Factors associated with compliance with physical activity recommendations among adolescents in Huesca. = Factors associated with compliance with physical activity recommendations among adolescents in Huesca. Rev Psicol del Deport. 2015;24:147–54.

Patrick K, Sallis JF, Prochaska JJ, et al. A multicomponent program for nutrition and physical activity change in primary care: Pace+ for adolescents. Arch Pediatr Adolesc Med. 2001;155:940–6.

Resaland GK, Aadland E, Moe VF, Aadland KN, Skrede T, Stavnsbo M, et al. Effects of physical activity on schoolchildren’s academic performance: The Active Smarter Kids (ASK) cluster-randomized controlled trial. Prev Med (Baltim). 2016;91:322–8.

Riley N, Lubans D, Holmes K, Morgan P. Findings From the EASY Minds Cluster Randomized Controlled Trial: Evaluation of a Physical Activity Integration Program for Mathematics in Primary Schools. J Phys Act Heal. 2016;13:198–206.

Riley N, Lubans DR, Holmes K, Morgan PJ. Rationale and study protocol of the EASY Minds (Encouraging Activity to Stimulate Young Minds) program: cluster randomized controlled trial of a primary school-based physical activity integration program for mathematics. BMC Public Health. 2014;14:816.

Rosenberg DE, Norman GJ, Sallis JF, Calfas KJ, Patrick K. Covariation of adolescent physical activity and dietary behaviors over 12 months. J Adolesc Health. 2007;41:472–8.

Routen A, Zhou D, Tymms P, Thomson K, Curtis S, Moore H, et al. Objectively measured physical activity and sedentary time and self-reported school travel mode in a population of English secondary school students ( 11-12 years ): The MOVE Project. 3:12.

Safdie M, Jennings-Aburto N, Lévesque L, Janssen I, Campirano-Núñez F, López-Olmedo N, et al. Impact of a school-based intervention program on obesity risk factors in Mexican children. Salud Publica Mex. 2013;55 SUPPL.3:S374–87.

Salmon J, Ball K, Crawford D, Booth M, Telford A, Hume C, et al. Reducing sedentary behaviour and increasing physical activity among 10-year-old children: Overview and process evaluation of the “Switch-Play” intervention. Health Promot Int. 2005;20:7–17.

Sebire SJ, Edwards MJ, Kesten JM, May T, Banfield KJ, Bird EL, et al. Process evaluation of the Bristol girls dance project. BMC Public Health. 2016;16:349.

Serra-Paya N, Ensenyat Solé A, Real Gatius J, Castro Viñuales I, Zapara Rojas A, Galindo Ortego G, et al. Evaluation of a family intervention programme for the treatment of overweight and obese children (Nereu Programme): a randomized clinical trial study protocol. BMC Public Health. 2013;13:1–29.

Siceloff ER, Wilson DK, Van Horn L. A Longitudinal Study of the Effects of Instrumental and Emotional Social Support on Physical Activity in Underserved Adolescents in the ACT Trial. Ann Behav Med. 2013;:1–9.

Sigmund E, Sigmundov?? D. Longitudinal 2-year follow-up on the effect of a non-randomised school-based physical activity intervention on reducing overweight and obesity of Czech children aged 10-12 years. Int J Environ Res Public Health. 2013;10:3667–83.

Smith JJ, Morgan PJ, Plotnikoff RC, Stodden DF, Lubans DR. Mediating effects of resistance training skill competency on health-related fitness and physical activity: the ATLAS cluster randomised controlled trial. J Sports Sci. 2016;34:772–9.

Stevens J, Suchindran C, Ring K, Baggett CD, Jobe JB, Story M, et al. Physical activity as a predictor of body composition in American Indian children. Obes Res. 2004;12:1974–80.

Stockton MB, McClanahan BS, Lanctot JQ, Klesges RC, Beech BM. Identification of facilitators and barriers to participation in weight gain prevention research by African American girls. Contemp Clin Trials. 2012;33:38–45.

Stone EJ, Norman JE, Davis SM, Stewart D, Clay TE, Caballero B, et al. Design, implementation, and quality control in the Pathways American-Indian multicenter trial. Prev Med (Baltim). 2003;37 SUPPL. 1.

Suchert V, Isensee B, Hansen J, Johannsen M, Krieger C, Müller K, et al. “ lauft.” - a school-based multi-component program to establish a physically active lifestyle in adolescence: Study protocol for a cluster-randomized controlled trial. Trials. 2013;14:416.

Sutherland R, Campbell E, Lubans DR, Morgan PJ, Okely AD, Nathan N, et al. A cluster randomised trial of a school-based intervention to prevent decline in adolescent physical activity levels: study protocol for the “Physical Activity 4 Everyone” trial. BMC Public Health. 2013;13:57.

Taylor RW, Mcauley K a, Barbezat W, Farmer VL, Williams SM, Mann JI. Two-year follow-up of an obesity prevention initiative in children : the APPLE project. 2008.

Toftager M, Christiansen LB, Kristensen PL, Troelsen J. SPACE for physical activity--a multicomponent intervention study: study design and baseline findings from a cluster randomized controlled trial. BMC Public Health. 2011;11:777.

Van Lippevelde W, Bere E, Verloigne M, van Stralen MM, De Bourdeaudhuij I, Lien N, et al. The role of family-related factors in the effects of the UP4FUN school-based family-focused intervention targeting screen time in 10- to 12-year-old children: the ENERGY project. BMC Public Health. 2014;14:857.

Van Stralen MM, Te Velde SJ, Van Nassau F, Brug J, Grammatikaki E, Maes L, et al. Weight status of European preschool children and associations with family demographics and energy balance-related behaviours: A pooled analysis of six European studies. Obes Rev. 2012;13 SUPPL. 1:29–41.

Verloigne M, Ahrens W, De Henauw S, Verbestel V, Mårild S, Pigeot I, et al. Process evaluation of the IDEFICS school intervention: Putting the evaluation of the effect on children’s objectively measured physical activity and sedentary time in context. Obes Rev. 2015;16 December:89–102.

Verstraete, S., Cardon, G., De Clercq, D., & De Bourdeaudhuij I. Effectiveness of a two-year health-related physical education intervention in elementary schools. J Teach Phys Educ. 2007;26:20–34.

Vik FN, Lien N, Berntsen S, De Bourdeaudhuij I, Grillenberger M, Manios Y, et al. Evaluation of the UP4FUN intervention: A cluster randomized trial to reduce and break up sitting time in European 10-12-year-old children. PLoS One. 2015;10:1–15.

Williden M, Taylor RW, McAuley KA, Simpson JC, Oakley M, Mann JI. The APPLE Project: An Investigation of the Barriers and Promoters of Healthy Eating and Physical Activity in New Zealand Children Aged 5-12 Years. Health Educ J. 2006;65:135–48.

Wilson DK, Kitzman-Ulrich H, Williams JE, Saunders R, Griffin S, Pate R, et al. An overview of “The Active by Choice Today” (ACT) trial for increasing physical activity. Contemp Clin Trials. 2008;29:21–31.

Zahner L, Puder JJ, Roth R, Schmid M, Guldimann R, Pühse U, et al. A school-based physical activity program to improve health and fitness in children aged 6-13 years (“Kinder-Sportstudie KISS”): study design of a randomized controlled trial [ISRCTN15360785]. BMC Public Health. 2006;6:147.
